# Diabetes Mellitus and Prostate Cancer Risk—A Systematic Review and Meta-Analysis

**DOI:** 10.3390/cancers16234010

**Published:** 2024-11-29

**Authors:** Agnieszka Drab, Krystian Wdowiak, Wiesław Kanadys, Krzysztof Zajączkowski, Paweł Koczkodaj, Urszula Religioni, Mariola Borowska, Magdalena Łoś, Macarena Lozano-Lorca

**Affiliations:** 1 Chair of Preclinical Sciences, Department of Medical Informatics and Statistics, Medical University of Lublin, 20-090 Lublin, Poland; agnieszkadrab@umlub.pl; 2 Faculty of Medicine, Medical University of Lublin, K. Jaczewskiego 5 Street, 20-090 Lublin, Poland; wieslaw.kanadys@wp.pl; 3 Department of Urology, Center of Oncology of the Lublin Region St. Jana z Dukli, 20-091 Lublin, Poland; biuro@onkocentrum.pl; 4 Department of Cancer Epidemiology and Primary Prevention, Maria Sklodowska-Curie National Research Institute of Oncology, 02-781 Warsaw, Poland; pawel.koczkodaj@nio.gov.pl (P.K.); mariola.borowska@nio.gov.pl (M.B.); 5 School of Public Health, Centre of Postgraduate Medical Education of Warsaw, 00-041 Warsaw, Poland; urszula.religioni@gmail.com; 6 Department of Social Medicine and Public Health, Warsaw Medical University, 02-007 Warsaw, Poland; mbogdan@wum.edu.pl; 7 Departamento de Medicina Preventiva y Salud Pública, Universidad de Granada, 18071 Granada, Spain; macarenalozano@ugr.es; 8 Instituto de Investigación Biosanitaria ibs, GRANADA, 18012 Granada, Spain; 9 Consorcio Centro de Investigación Biomédica en Red de Epidemiología y Salud Pública (CIBERESP), 28029 Madrid, Spain

**Keywords:** diabetes mellitus, prostate cancer, meta-analysis

## Abstract

Introduction: Prostate cancer is the second most commonly diagnosed malignant tumor worldwide and poses a significant challenge to public health. This systematic review and meta-analysis aims to investigate the association between diabetes mellitus and the risk of developing prostate cancer. Methods: We conducted a search of PubMed, Embase, and the Cochrane Library from 1998 to 2024. The risk of bias within the included studies was assessed using the Newcastle–Ottawa Scale. The DerSimonian–Laird random-effect model was employed for the meta-analysis. Heterogeneity was evaluated using a forest plot and statistically assessed via the Q test, I^2^ index, and *p*-values. Results: Forty-three studies involving a total of 3,746,769 patients were included. Both case–control (pOR = 0.68, 95% CI: 0.61–0.97; I^2^ = 92.24%) and cohort studies (pRR = 0.71, 95% CI: 0.59–0.99; I^2^ = 85.41%) suggest that diabetes mellitus is associated with a reduced risk of prostate cancer, though with significant heterogeneity (*p* < 0.05). Subgroup analysis revealed that the risk of developing prostate cancer was significantly higher in patients with a family history of prostate cancer (pRR = 1.25, 95% CI: 1.16–1.35; I^2^ = 69.51%). Conclusions: Our meta-analysis of recent observational studies indicates that diabetes mellitus is associated with a reduced risk of developing prostate cancer.

## Simple Summary

The incidence of prostate cancer is increasing globally. The aim of this meta-analysis was examine the association between diabetes and risk of prostate cancer. We systematically searched PubMed, EMBASE, and the Cochrane Library databases to identify eligible articles. A total of 41 articles were included in this study. The results showed that risk of prostate cancer decreased in diabetes patients (case–control studies: pOR = 0.68, 95% CI: 0.61–0.97; I^2^ = 92.24%; cohort studies: pRR = 0.71, 95% CI: 0.59–0.99; I^2^ = 85.41%). Our meta-analysis suggests a protective effect against prostate cancer risk.

## 1. Introduction

Prostate cancer is the second most diagnosed malignant tumor worldwide and the most frequently diagnosed cancer among men in numerous countries, particularly in the Americas, Western Europe, and Australia [1,2,3]. It remains a leading cause of cancer-related deaths among men in at least a quarter of the world’s nations, notably in the Caribbean, parts of South America, and much of Africa [1]. The variations in prostate cancer incidence and mortality rates are primarily attributed to disparities in access to screening, diagnosis, and effective treatment [2,4].

Risk factors for prostate cancer can be categorized as non-modifiable and modifiable [1]. Non-modifiable factors include age, family history, genetic predisposition, and height [1]. Age is a well-documented risk factor, with the likelihood of developing prostate cancer significantly increasing after the age of 60 [4,5]. A family history of prostate cancer and genetic predisposition also contribute to heightened risk; men with a father or brother diagnosed with the disease face an approximate 2.5-fold increase in their own risk [6,7]. It is estimated that genetic factors count for around 60% of prostate cancer cases [8,9]. Additionally, having a first-degree relative with breast cancer elevates the risk of prostate cancer by about 1.2 times [10]. Although androgenic alopecia was previously thought to be a risk factor, several meta-analyses have found no significant association [11,12]. Taller stature appears to predispose men to both prostate cancer and a more aggressive disease course, potentially linked to insulin-like growth factors during adolescence, although the underlying mechanisms remain unclear [13].

Modifiable risk factors include physical activity, diet, alcohol consumption, and smoking [1]. The impact of physical activity on prostate cancer risk remains uncertain; while many studies report no significant effect on the development of the disease [14,15], others have associated low levels of physical activity with more severe outcomes [16,17]. Numerous studies have examined the relationship between diet and prostate cancer, with evidence suggesting that certain dietary patterns may offer protective effects [1]. The Mediterranean diet [18], vegetarian diets [19], and foods rich in phytoestrogens [20,21] have been linked to a lower risk of prostate cancer, while inflammatory and hyperinsulinemic diets [22], high dairy protein intake [23], and trans-fatty acids [24] have been associated with an increased risk. Recent studies suggest that alcohol consumption does not significantly influence prostate cancer risk [25], whereas smoking and the use of smokeless tobacco increase both the risk and severity of the disease [26,27].

Diabetes is a prevalent global condition [28], affecting an estimated 537 million people, nearly half of whom are unaware of their condition [29]. Type 1 diabetes is primarily associated with autoimmune processes [30], while type 2 diabetes arises from a combination of genetic factors, obesity, and a sedentary lifestyle [31], accounting for approximately 90% of all diabetes cases [28]. Diabetes is also a recognized risk factor for several cancers [32]. Thus, type 1 diabetes has been linked to an increased risk of cancers of the liver, pancreas, kidneys, esophagus, stomach, lungs, and thyroid, as well as squamous cell carcinoma and leukemia [33,34,35,36]. In contrast, type 2 diabetes is associated with an elevated risk of hepatocellular, biliary tract, gallbladder, pancreatic, gastrointestinal, kidney, bladder, lung, thyroid, breast, ovarian, endometrial, and oral cancers, as well as leukemia, glioma, and melanoma [32,37,38,39]. The mechanisms by which diabetes predisposes individuals to cancer are not fully understood [40]; however, researchers suggest that insulin-like growth factors (IGF-1 and IGF-2) and the stimulating effects of hyperglycemia on cancer cell proliferation may play significant roles [41,42,43].

Interestingly, some studies indicate that diabetes may have a protective effect against certain cancers, including those of the brain, buccal cavity, esophagus, lungs, breast, bladder, and larynx [38]. The relationship between diabetes and prostate cancer has been extensively researched, with some studies indicating that diabetes may reduce the risk of developing this cancer [1]. However, conflicting findings from various studies have complicated the ability to draw definitive conclusions [1]. Therefore, the aim of this meta-analysis is to investigate the association between diabetes and the risk of prostate cancer.

## 2. Materials and Methods

### 2.1. Selection Criteria

This systematic review and meta-analysis was designed according to Preferred Reporting Items for Systematic Reviews and Meta-analysis (PRISMA) guidelines [44]. This protocol was previously registered on the Prospero System (ID: CRD42024524461).

### 2.2. Data Sources and Search Strategy

We identified eligible observational studies published from January 1998 up to January 2024 in Pubmed, Embase, and the Cochrane Library. Research papers were selected using a combination of the following keywords: “diabetes” OR “diabetes mellitus”; AND “prostate cancer” OR “prostate neoplasm”; AND “risk”. All abstracts obtained from our search were reviewed for relevance, and we manually examined bibliographies and review articles for additional citations and obtained the full text of all potentially relevant articles. Unpublished reports were not considered.

### 2.3. Study Selection

The systematic review included studies that met the following criteria: (i) cohort or case–control study design; (ii) conducted on human subjects; (iii) published in English; (iv) reporting effect measures [hazard ratios (HRs), risk ratios (RRs) or odds ratios (ORs)] with 95% confidence intervals (95% CIs), or providing sufficient data to calculate them; (v) full-text availability.

Studies were excluded for the following reasons: (i) no raw data available (reporting no effect measures [hazard ratios (HRs), risk ratios (RRs) or odds ratios (ORs)] with 95% confidence intervals (95% CIs), or no sufficient data to calculate them; (ii) duplicate studies; (iii) inappropriate study designs such as descriptive studies, reviews, systematic reviews, meta-analyses, experimental studies, editorials, letters, conferences, abstracts, or theses.

Two independent reviewers (A. Drab, W. Kanadys) conducted the search and identified eligible articles. Initially, they screened the titles and abstracts to determine which articles qualified for full-text screening. The two reviewers critically examined the full text of the articles based on the study eligibility criteria. Whenever there was a disagreement as to which article was to be included for full title and abstract screening as well as for full paper review, this was resolved through discussion. Consultations with co-authors took place on 15–16 February 2024.

### 2.4. Study Quality Assessment

The Newcastle–Ottawa Scale (NOS) was used to assess the quality of the included studies [45]. Two authors independently performed quality assessment (A. Drab, W. Kanadys). In the methodological evaluation process, scores from 0 to 3, from 4 to 6, and from 7 to 9 were given for low, medium, and high quality, respectively. The NOS comprises 8 items, which fall into three domains: selection of the population (ranging from 0 to 4 points), comparability of the groups (ranging from 0 to 2 points), and assessment of the outcome (ranging from 0 to 3 points). We considered a study to be of high quality when the total score was ≥7 points.

### 2.5. Statistical Analysis

We examined the relationship between diabetes mellitus and prostate cancer risk by analyzing relative risks (RRs) for cohort studies and odds ratios (ORs) for case–control studies, with 95% confidence intervals (CIs). Hazard ratios (HRs) from some studies were converted into RRs, as prostate cancer is a relatively rare event, allowing rate ratios to be treated as comparable to RRs [46,47].

To account for variability between studies, we used a DerSimonian–Laird random-effect model [48]. Heterogeneity was assessed visually using forest plots and statistically with the Q test, I^2^ index, and *p*-value. Heterogeneity was classified as high (I^2^ > 75%), moderate (I^2^ = 50–75%), or low (I^2^ < 50%).

Subgroup analyses were conducted to identify sources of heterogeneity, focusing on factors like family history of prostate cancer, obesity, hypertension, smoking, and alcohol consumption. Publication bias was assessed using funnel plots, Begg’s test [49], and Egger’s test [50]. The meta-analysis was performed using the STATISTICA 13.3 software (StatSoft, Kraków, Poland).

## 3. Results

**Figure 1:**
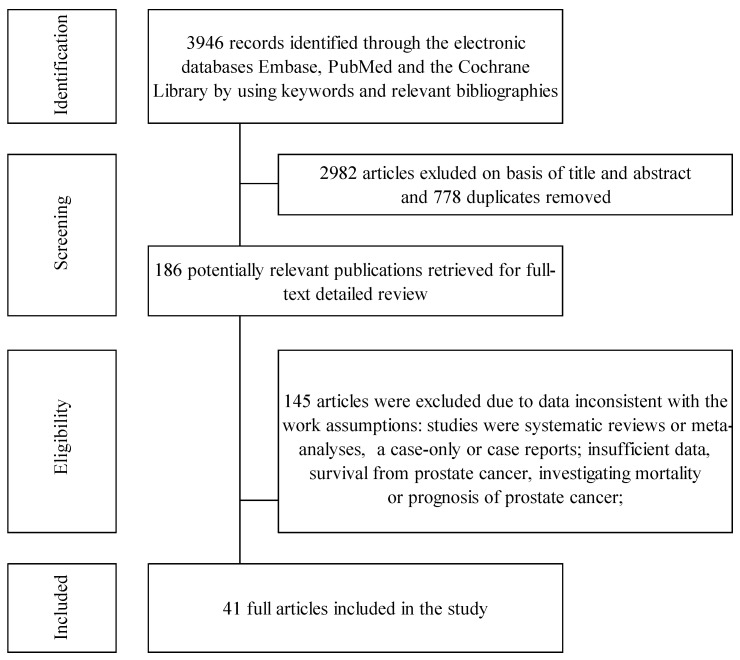
Flowchart of the selection procedure for included studies.

We retrieved a total of 3946 reference records. After excluding 778 duplicates, 2982 records were removed for not aligning with the objectives of this review. A further 145 references did not meet the eligibility criteria. Finally, we included 41 publications, comprising 43 studies in the meta-analysis. Of these studies, 23 were cohort studies and 20 were case–control studies. A flowchart illustrating the study selection process is shown in Figure 1.

Of the 41 publications (43 studies) [51,52,53,54,55,56,57,58,59,60,61,62,63,64,65,66,67,68,69,70,71,72,73,74,75,76,77,78,79,80,81,82,83,84,85,86,87,88,89,90,91] included in the analysis, 20 studies were conducted in the USA, 13 in Europe, 6 in Asia, 2 as multicenter studies (Europe and the United States), and 1 each in Australia and Canada. The main characteristics of the studies included in the meta-analysis are presented in Table 1 for both cohort and case–control studies. In total, these 43 studies involved 2,668,356 patients in the cohort/control groups and 124,823 prostate cancer cases. The NOS quality points ranged between 5 and 9, and the average score was 7.24 for included studies.

**Figure 2:**
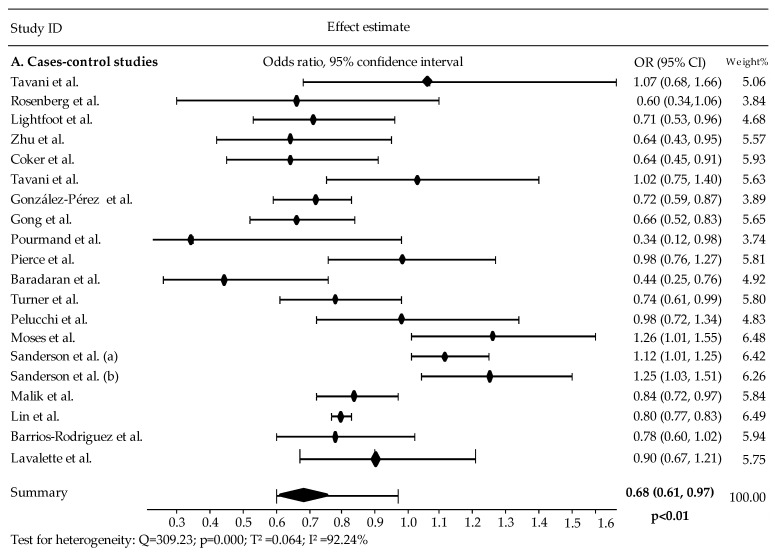

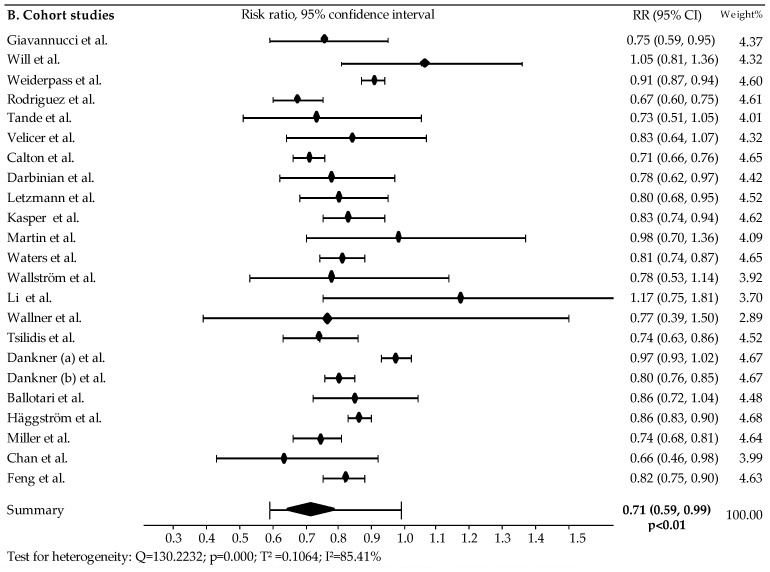
Forest plot of risk ratio (RR) and odds ratio (OR) estimates of prostate cancer comparing diabetes. Abbreviations: Sanderson et al.’s (**A**) Study on Black Men; Sanderson et al.’ (**B**) Study on White Men; Dankner et al. on (**A**) incident diabetes; Dankner et al. on (**B**) prevalent diabetes [51,52,53,54,55,56,57,58,59,60,61,62,63,64,65,66,67,68,69,70,71,72,73,74,75,76,77,78,79,80,81,82,83,84,85,86,87,88,89,90,91].

Case–control studies (Figure 2A) suggest that diabetes mellitus may be associated with a reduced risk of prostate cancer, with a pooled odds ratio (pOR) of 0.68 (95% CI: 0.61–0.97) and high heterogeneity (I^2^ = 92.24%; *p* for heterogeneity < 0.05). Similarly, cohort studies (Figure 2B) indicate a comparable reduction in prostate cancer risk, with a pooled relative risk (pRR) of 0.71 (95% CI: 0.59–0.99), though also with significant heterogeneity (I^2^ = 85.41%; *p* for heterogeneity < 0.05).

In order to deepen the analysis of the problem, an additional subgroup analysis was performed. The risk of prostate cancer developing was significantly higher in patients with a family history of prostate cancer (1.25, 95% CI = 1.16–1.35, *p* < 0.001). Differences between patients with obesity and hypertension and men who were drinking alcohol and cigarette smoking and those without and who did not turned out to be statistically insignificant. The results of the stratified subgroup analysis are summarized in Table 2.

## 4. Discussion

In recent years, numerous scientific studies have indicated a reduced risk of developing prostate cancer in men with diabetes, though opposing results have been found in studies focusing on Asian populations [40,92,93,94,95,96,97]. In the meta-analysis conducted by Zhang et al. [92], which included 12 case–control studies and 25 cohort studies, a negative correlation between diabetes and prostate cancer was confirmed only in population-based studies (RR = 0.719), in U.S. cohort studies (RR = 0.789), and in studies where the observation period exceeded 5 years (RR = 0.784) and 10 years (RR = 0.912) [92]. In our study, the negative correlation between prostate cancer risk and diabetes was confirmed in both case-control and cohort studies, most likely due to the inclusion of a larger number of case–control studies, allowing for a more thorough investigation of this issue.

A more recent analysis on this topic was conducted by Jian Gang et al. [96], who reviewed 24 case–control studies and 32 cohort studies. The RR for cohort studies was 0.86, that for case–control studies was 0.90, and overall it was 0.88. The results are consistent with ours, although the RR values in our study are slightly lower. The difference may be partly because Jian Gang et al. [96] did not assess the methodological quality of the articles included in the analysis, unlike our study. The most recent analysis on this subject was conducted by Ye et al. [40], which included 35 case–control studies, 31 cohort studies, and 6 cross-sectional studies. Although the number of articles included is greater than in our analysis, the authors also incorporated studies with lower NOS scores than we did. The analysis showed an OR of 0.89 when all studies were included and demonstrated that the negative correlation between diabetes and prostate cancer is typical for all populations except the Asian population.

In the meta-analysis conducted by Long et al. [93], four cohort studies and three case–control studies from the Asian population were taken into consideration. This analysis showed an increased risk of developing prostate cancer in individuals with diabetes (adjusted RR = 1.31). Our study includes one study from Long et al. [66] and four more recent studies on the Asian population [57,60,68,77]. The limitation of the study by Long et al. [93] is the small number of studies brought into consideration, so the result should be interpreted with caution. A positive correlation between diabetes and prostate cancer in the Asian population was also confirmed by the analysis by Jian Gang et al. [96] (RR = 1.72), though, again, the number of analyzed articles was small (seven), and some were used in the previous analysis [93]. The most recent analysis by Ye et al. [40] incorporated nine studies from Asia and also showed an increased risk of developing prostate cancer in individuals with diabetes (OR = 1.55). The meta-analysis by Xu et al. [94] included nine studies, which also appeared in our meta-analysis [28,67,69,74,75,76,79,83,84]. A limitation of this meta-analysis is the relatively small number of studies considered, but it demonstrated a previously unassessed negative association between diabetes and prostate cancer at all stages of the disease, with a stronger effect observed in the early (RR = 0.74) and localized (RR = 0.72) stages, compared to advanced stages (RR = 0.78) [94]. The meta-analysis by Huang et al. [95] investigated the relationship between prediabetes and the risk of developing various cancers. No association was found; however, the analysis had only three cohort studies, so the results should be interpreted with caution.

Our subgroup analysis showed that a family history of prostate cancer significantly increased the risk of developing this malignancy, and is consistent with the findings of previous studies [6,7]. However, the subgroup analysis did not find an association between obesity and prostate cancer, although this observation should be treated with caution due to the inclusion of a small number of studies. The results of other studies linking these two conditions are inconclusive: some authors associate obesity only with worse prognosis in prostate cancer, while others also suggest its role in carcinogenesis [98,99]. These observations are linked both to lower PSA levels in obese individuals, leading to later detection of the disease, and to the direct effect of substances secreted by adipocytes on cancer cell growth [98,99]. Our analysis did not show a link between hypertension and prostate cancer risk, which is confirmed by other studies [100]. The same applies to alcohol consumption [25]. However, the case is different for tobacco smoking, as other studies suggest that tobacco smoking increases the risk of prostate cancer and worsens the course of the disease [26,27]. The molecular mechanisms explaining the relationship between diabetes and prostate cancer risk are not yet fully understood [40]. One potential cause of this phenomenon is the relative insulin deficiency observed in long-standing diabetes, which leads to lower levels of C-peptide and IGF-1 compared to healthy individuals [54]. This mechanism appears highly plausible, particularly in light of the positive correlation between IGF-1 levels and prostate cancer risk, as documented in numerous scientific studies [101]. It is also worth noting that in vitro studies have demonstrated that insulin acts as a growth-stimulating factor for prostate epithelial cells, and its elevated levels are associated with a higher risk of both the development and recurrence of prostate cancer. This provides another possible explanation for how the relative insulin deficiency seen in long-standing diabetes might lead to a decreased risk of prostate cancer [79]. Some researchers further highlight the role of genetic factors in this association. For example, one study found that ERG-positive tumors showed increased expression of both insulin and IGF-1 receptors compared to ERG-negative tumors [102]. Additionally, experimental studies have suggested that PTEN mutations may reduce the risk of type 2 diabetes by enhancing insulin sensitivity [103]. Long-standing type 2 diabetes is also known to lead to a decrease in testosterone levels in the blood. This reduction is likely a consequence of worsening insulin resistance, a decline in the number of Leydig cells, and disruptions in testosterone secretion. Lower testosterone levels are generally considered to be a factor that does not favor the proliferation of prostate cells [104,105]. However, it should be noted that the current body of literature provides mixed evidence on the relationship between testosterone levels and prostate cancer risk. While some studies confirm a connection between elevated testosterone levels and a higher risk of prostate cancer, others suggest that this relationship is minimal or insignificant [79]. Furthermore, some research indicates that low levels of sex hormone-binding globulin (SHBG) may predispose individuals to prostate cancer, as reduced SHBG results in a greater availability of free testosterone in the body [79]. As the duration of diabetes increases, a declining ratio of testosterone to SHBG has been observed, which leads to a reduced availability of biologically active testosterone. High levels of active testosterone have been linked to an increased risk of developing prostate cancer [79]. The lower incidence of prostate cancer among diabetics has also been attributed to the disease’s impact on participation in PSA screening and PSA levels [54]. Unfortunately, the data on this topic are contradictory, as some studies have shown that diabetics participate in screening more frequently [60,106], while others suggest the opposite [107]. However, the association between diabetes and low PSA levels is well documented in the literature [108]. Given this, some researchers have highlighted the possibility of a negative correlation between diabetes and prostate cancer due to lower cancer detection rates in diabetics. Feng et al. [54] considered this factor in their meta-analysis, noting that while it explains part of the relationship between these conditions, it is not the only contributing factor.

Past research has also investigated the impact of diabetes medications on prostate cancer risk [109,110]. While metformin has been shown to have a preventive effect on the development of some cancers, this does not apply to prostate cancer [111,112]. Studies have also linked reduced prostate cancer risk to the use of other antidiabetic drugs, but none have shown a significant association [112]. A theory was previously proposed suggesting that the vascular changes typical of diabetes might prevent prostate cancer development [112], but this hypothesis has not been supported by molecular evidence [113]. Despite the strengths of this study, it is also important to acknowledge its limitations. First, we did not take into account the impact of different types of diabetes on cancer development such as diet, physical activity, stress, and others which has been addressed in numerous studies in the past and may lead to certain biases. Second, diabetes is associated with various factors that could influence the development of prostate cancer, but these were not considered in the study. Third, the current literature indicates that the association between diabetes and prostate cancer risk may vary depending on the duration and severity of the disease. Additionally, heterogeneity for the included studies was high, which could have influenced on the results, especially with respect to study design.

## 5. Conclusions

Our comprehensive meta-analysis of the latest observational studies provides further evidence of the association between diabetes and prostate cancer. Our study suggests that diabetes mellitus is associated with a decreased risk of prostate cancer. A major strength of our research is the inclusion of a larger number of studies compared to many other meta-analyses addressing this issue. It is also worth noting that meta-analyses which incorporated more studies than ours did not assess the quality of the studies or accepted studies with lower NOS scores than we did. However, more investigations are needed to understand the connection between diabetes mellitus and its associated comorbidities (such as obesity and hypertension) and prostate carcinogenesis. It would also be crucial to conduct studies that differentiate between types of diabetes, explore the molecular mechanisms underlying the association described in more detail, and investigate the relationship between prediabetic conditions and this cancer.

## Figures and Tables

**Table 1:** Characteristics of the studies included in the meta-analysis and systematic review.

First Author and Publication Year	Location and Study Period	Follow-Up Duration (Years)	No of PCa Cases	No of Cohort ^a^/ Control ^b^	NOS Score
Case–control studies					
Barrios-Rodriguez et al., 2022 [51]	Spain 2008–2013	NR	136	1379	8
Lavalette et al., 2022 [52]	France 2012–2013	NR	108	879	6
Lin et al., 2020 [53]	Sweden 2005–2016	8.5	4186	129,847	7
Malik et al., 2018 [54]	Pakistan 2010–2015	6	234	886	8
Sanderson et al., 2013 [55]	United States 2002–2009 Study on Black Men	7	4283	18,809	6
	United States 2002–2009 Study on White Men	7	1913	6404	6
Moses et al., 2012 [56]	USA 2000–2009	8	369	483	7
Pelucchi et al., 2011 [57]	Italy 1991–2002	NR	1451	1294	6
Turner et al., 2010 [58]	United Kingdom 2002–2006	4	1966	6479	8
Baradaran et al., 2009 [59]	Iran 2005–2009	2	317	194	6
Pierce et al., 2008 [60]	United States 1993–1996	5	1644	1752	7
Pourmand et al., 2007 [61]	Iran 2005–2007	3	75	130	7
Gong et al., 2006 [62]	United States 1996–2003	7	10,258	1936	6
Gonzales-Peres et al., 2005 [63]	Spain 1995–2001	6	2183	12,183	7
Tavani et al., 2005 [64]	Italy 1991–2002	8.2	1294	2745	6
Coker et al., 2004 [65]	USA 1992–2001	NR	72	760	6
Lightfoot et al., 2004 [66]	Canada 1995–1999	7	760	2392	5
Zhu et al., 2004 [67]	United States 1982–1995	11	1110	2200	5
Rosenberg et al., 2002 [68]	USA 1984–1986	10	320	509	7
Tavani et al., 2002 [69]	Italy, Greece 1983–1997	8	608	1616	5
Cohort Studies					
Miller et al., 2021 [70]	Multicenter * 2004–2015	7	11,469	136,083	6
Feng et al., 2020 [71]	United States 1986–2014	28	6733	49,392	8
Chan et al., 2019 [72]	Australia 1996–2004	10	315	3149	7
Ballotari et al., 2017 [73]	Italy 2010–2013	4	134	186,931	7
Häggström et al., 2017 [74]	Sweden 2006–2013	5	23,882	612,846	7
Dankner et al., 2016 [75]	Israel 2002–2012 incident diabetes	11	2713	183,835	6
	Israel 2002–2012 prevalent diabetes	11	1672	74,756	6
Tsilidis et al., 2015 [76]	Multicenter * 1992–2000	12	4531	139,131	6
Wallner et al., 2011 [77]	United States 1990–2005	15	206	2115	7
Li Q. et al., 2010 [78]	Japan 1995–2003	8	230	22,458	9
Kasper et al., 2009 [79]	United States 1986–2004	18	4511	51,529	8
Martin et al., 2009 [80]	Norway 1995–1997	9.3	687	29,364	9
Wallström et al., 2009 [81]	Sweden 1991–1996	11	817	10,564	7
Waters et al., 2009 [82]	United States 1993–2005	8	5941	86,303	8
Darbinian et al., 2008 [83]	United States 1964–1973	18.4	2833	47,209	9
Leitzmann et al., 2008 [84]	United States 1993–2001	8.9	2085	33,088	8
Calton et al., 2007 [85]	United States 1995–2000	5	11,193	328,316	8
Velicer et al., 2007 [86]	United States 2000–2002	2	827	35,239	9
Tande et al., 2006 [87]	United States 1987–2000	12.1	385	6429	9
Rodriguez et al., 2005 [88]	United States 1992–2001	9	5318	72,670	7
Weiderpass et al., 2002 [89]	Sweden 1965–1994	NR	2455	13,595	6
Will et al., 1999 [90]	United States 1959–1960	5	2523	305,065	6
Giovannucci 1998 [91]	United States 1986–1994	8	76	46,412	5

* Europe and United States (EPIC study); ^a^ for cohort studies; ^b^ for case–control studies; NR—not reported.

**Table 2:** Stratified analysis on the association between diabetes and the risk of prostate cancer.

Covariates		No. of Studies	RR (95% CI)	I^2^	*p*<
Family history of prostate cancer	yes	12	1.25 (1.16, 1.35)	69.51%	0.001
	no	12			
Study design	case–control	20	0.87 (0.74, 0.91)	73.35%	0.266
	cohort	23			
Obesity	BMI: ≤25 kg/m^2^	5	1.03 (0.97, 1.10)	00.00%	0.328
	BMI: ≥30 kg/m^2^	5			
Hypertension	yes	6	1.03 (0.95, 1.12)	63.82%	0.503
	no	6			
Cigarette smoking	yes	9	1.02 (0.94, 1.10)	66.54%	0.642
	no	9			
Drinking alcohol	yes	6	0.91 (0.80, 1.04)	66.54%	0.555
	no	6			
